# 1-(6-Methyl-4-phenyl-2-sulfanyl­idene-1,2,3,4-tetrahydro­pyrimidin-5-yl)ethanone

**DOI:** 10.1107/S1600536810031296

**Published:** 2010-08-11

**Authors:** N. Anuradha, A. Thiruvalluvar, S. Chitra, K. Pandiarajan, R. J. Butcher

**Affiliations:** a PG Research Department of Physics, Rajah Serfoji Government College (Autonomous), Thanjavur 613 005, Tamil Nadu, India; b Department of Chemistry, Annamalai University, Annamalai Nagar 608 002, Tamilnadu, India; c Department of Chemistry, Howard University, 525 College Street NW, Washington, DC 20059, USA

## Abstract

In the title compound, C_13_H_14_N_2_OS, the heterocyclic ring adopts a flattened boat conformation with the plane through the four coplanar atoms making a dihedral angle of 86.90 (13)° with the phenyl ring, which adopts an axial orientation. The thionyl, acetyl and methyl groups all have equatorial orientations. Inter­molecular N—H⋯O, N—H⋯S and C—H⋯O hydrogen bonds are found in the crystal structure.

## Related literature

For chemical and biological applications of dihydro­pyrimidinone derivatives, see: Chitra *et al.* (2010 ▶). For their applications and for related structures, see: Anuradha *et al.* (2008 ▶, 2009*a* ▶,*b* ▶,*c* ▶).
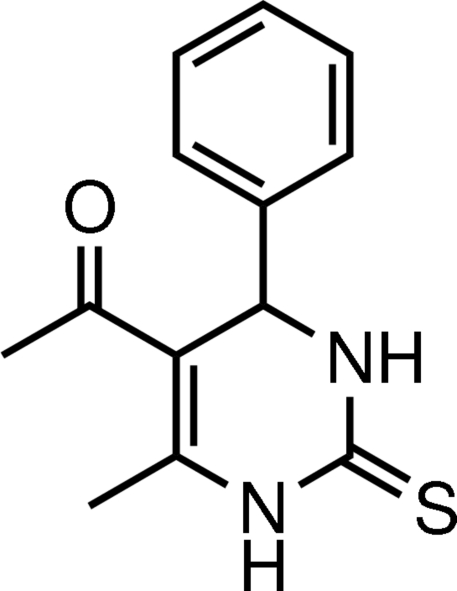


## Experimental

### 

#### Crystal data

C_13_H_14_N_2_OS
*M* *_r_* = 246.33
Monoclinic, 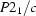

*a* = 7.8849 (10) Å
*b* = 7.2054 (5) Å
*c* = 21.555 (3) Å
β = 94.401 (12)°
*V* = 1221.0 (2) Å^3^
*Z* = 4
Cu *K*α radiation
μ = 2.23 mm^−1^
*T* = 295 K
0.44 × 0.31 × 0.16 mm

#### Data collection

Oxford Diffraction Xcalibur Ruby Gemini diffractometer
Absorption correction: multi-scan (*CrysAlis PRO*; Oxford Diffraction, 2010 ▶) *T*_min_ = 0.307, *T*_max_ = 1.000
4776 measured reflections
2531 independent reflections
2226 reflections with *I* > 2σ(*I*)
*R*_int_ = 0.029

#### Refinement

*R*[*F* ^2^ > 2σ(*F* ^2^)] = 0.081
*wR*(*F* ^2^) = 0.236
*S* = 1.22
2531 reflections
164 parameters
H atoms treated by a mixture of independent and constrained refinement
Δρ_max_ = 0.62 e Å^−3^
Δρ_min_ = −0.30 e Å^−3^

### 

Data collection: *CrysAlis PRO* (Oxford Diffraction, 2010 ▶); cell refinement: *CrysAlis PRO*; data reduction: *CrysAlis PRO*; program(s) used to solve structure: *SIR2002* (Burla *et al.*, 2003 ▶); program(s) used to refine structure: *SHELXL97* (Sheldrick, 2008 ▶); molecular graphics: *ORTEP-3* (Farrugia, 1997 ▶); software used to prepare material for publication: *PLATON* (Spek, 2009 ▶).

## Supplementary Material

Crystal structure: contains datablocks global, I. DOI: 10.1107/S1600536810031296/hg2697sup1.cif

Structure factors: contains datablocks I. DOI: 10.1107/S1600536810031296/hg2697Isup2.hkl

Additional supplementary materials:  crystallographic information; 3D view; checkCIF report

## Figures and Tables

**Table 1:** Hydrogen-bond geometry (Å, °)

*D*—H⋯*A*	*D*—H	H⋯*A*	*D*⋯*A*	*D*—H⋯*A*
N1—H1⋯O15^i^	0.80 (6)	2.14 (6)	2.898 (5)	158 (5)
N3—H3⋯S2^ii^	0.88 (4)	2.57 (4)	3.436 (4)	168 (4)
C61—H61*B*⋯O15^i^	0.96	2.54	3.333 (6)	140

Symmetry codes: (i) 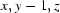; (ii) 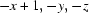.

**Fig. 1.**
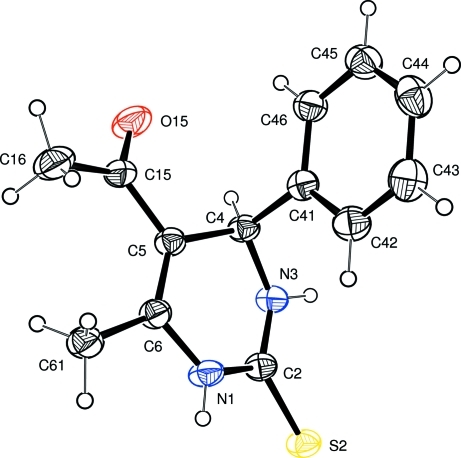
The molecular structure of the title compound, showing the atom-numbering scheme and displacement ellipsoids drawn at the 30% probability level. H atoms are shown as small spheres of arbitrary radius.

**Fig. 2.**
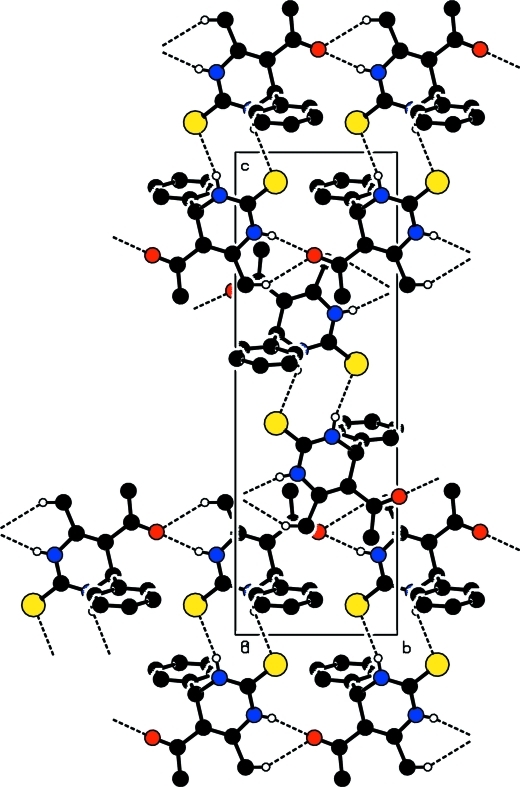
The packing of the title compound, viewed down the a axis. Dashed lines indicate hydrogen bonds. H atoms not involved in hydrogen bonding have been omitted.

## supplementary crystallographic information

### Comment

Dihydropyrimidinone derivatives exhibit a wide range of biological effects such as antibacterial and antifungal activities (Chitra *et al.*, 2010). The crystal structures of four very closely related compounds have recently been reported [Anuradha *et al.*, (2008, 2009*a*,*b*,*c*)]. This study of the title compound, was undertaken to compare the biological activity and structure of dihydropyrimidin-2(1*H*)-thione and its corresponding 2(1*H*)-one (Anuradha *et al.*, 2008).

In the title molecule, C_13_H_14_N_2_OS, Fig.1., the heterocyclic ring adopts a flattened boat conformation with the plane through the four coplanar atoms (C2,N3,C5,C6) making a dihedral angle of 86.90 (13)° with the phenyl ring, which adopts an axial orientation. The thionyl, acetyl and methyl groups all have equatorial orientations. Intermolecular N1—H1···O15, N3—H3···S2 and C61—H61B···O15 hydrogen bonds are found in the crystal structure (Fig. 2, Table 1).

### Experimental

A solution of acetylacetone (1.0012 g, 0.01 mol), benzaldehyde (1.06 g, 0.01 mol) and thiourea (1.149 g, 0.015 mol) was heated under reflux in the presence of calcium fluoride (0.0780 g, 0.001 mol) for 2 h (monitored by TLC). After completion of the reaction, the reaction mixture was cooled to room temperature and poured into crushed ice. The crude product containing also the catalyst was collected on a Buchner funnel by filtration. The mixture of the product and the catalyst was digested in methanol (40 ml). The undissolved catalyst was removed by filtration. The crude product was obtained by evaporation of methanol and further purified by recrystallization from hot ethanol to afford the pure title compound. Yield 96% (2.8 g).

### Refinement

The two N-bound H atoms were located in a difference Fourier map and refined freely; N1—H1 = 0.80 (6)Å and N3—H3 = 0.88 (4) Å. The remaining H atoms were positioned geometrically and allowed to ride on their parent atoms, with C*sp*^2^—H = 0.93, *C*(methyl)—H = 0.96, and C(methine)—H = 0.98 Å; *U*_iso_(H) = k*U*_eq_(C), where k = 1.5 for methyl and 1.2 for all other H atoms.

### Crystal data

**Table:**

C_13_H_14_N_2_OS	*F*(000) = 520
*M**_r_* = 246.33	*D*_x_ = 1.340 Mg m^−^^3^
Monoclinic, *P*2_1_/*c*	Melting point: 523.5 K
Hall symbol: -P 2ybc	Cu *K*α radiation, λ = 1.54184 Å
*a* = 7.8849 (10) Å	Cell parameters from 3041 reflections
*b* = 7.2054 (5) Å	θ = 5.6–77.1°
*c* = 21.555 (3) Å	µ = 2.23 mm^−^^1^
β = 94.401 (12)°	*T* = 295 K
*V* = 1221.0 (2) Å^3^	Prism, colourless
*Z* = 4	0.44 × 0.31 × 0.16 mm

### Data collection

**Table:**

Oxford Diffraction Xcalibur Ruby Gemini diffractometer	2531 independent reflections
Radiation source: Enhance (Cu) X-ray Source	2226 reflections with *I* > 2σ(*I*)
graphite	*R*_int_ = 0.029
Detector resolution: 10.5081 pixels mm^-1^	θ_max_ = 77.3°, θ_min_ = 5.6°
ω scans	*h* = −9→9
Absorption correction: multi-scan (*CrysAlis PRO*; Oxford Diffraction, 2010)	*k* = −5→9
*T*_min_ = 0.307, *T*_max_ = 1.000	*l* = −24→27
4776 measured reflections	

### Refinement

**Table:**

Refinement on *F*^2^	Primary atom site location: structure-invariant direct methods
Least-squares matrix: full	Secondary atom site location: difference Fourier map
*R*[*F*^2^ > 2σ(*F*^2^)] = 0.081	Hydrogen site location: inferred from neighbouring sites
*wR*(*F*^2^) = 0.236	H atoms treated by a mixture of independent and constrained refinement
*S* = 1.22	*w* = 1/[σ^2^(*F*_o_^2^) + (0.0912*P*)^2^ + 1.9142*P*] where *P* = (*F*_o_^2^ + 2*F*_c_^2^)/3
2531 reflections	(Δ/σ)_max_ = 0.001
164 parameters	Δρ_max_ = 0.62 e Å^−^^3^
0 restraints	Δρ_min_ = −0.30 e Å^−^^3^

### Special details

**Table:**

Geometry. Bond distances, angles *etc*. have been calculated using the rounded fractional coordinates. All su's are estimated from the variances of the (full) variance-covariance matrix. The cell e.s.d.'s are taken into account in the estimation of distances, angles and torsion angles
Refinement. Refinement of *F*^2^ against ALL reflections. The weighted *R*-factor *wR* and goodness of fit *S* are based on *F*^2^, conventional *R*-factors *R* are based on *F*, with *F* set to zero for negative *F*^2^. The threshold expression of *F*^2^ > 2σ(*F*^2^) is used only for calculating *R*-factors(gt) *etc*. and is not relevant to the choice of reflections for refinement. *R*-factors based on *F*^2^ are statistically about twice as large as those based on *F*, and *R*- factors based on ALL data will be even larger.

### Fractional atomic coordinates and isotropic or equivalent isotropic displacement parameters (Å^2^)

**Table:**

	*x*	*y*	*z*	*U*_iso_*/*U*_eq_	
S2	0.49206 (14)	−0.24803 (15)	0.06167 (5)	0.0540 (4)	
O15	0.7124 (6)	0.5124 (5)	0.21330 (16)	0.0771 (13)	
N1	0.6473 (5)	−0.1160 (5)	0.16583 (16)	0.0512 (11)	
N3	0.5890 (4)	0.0977 (5)	0.08897 (16)	0.0456 (10)	
C2	0.5819 (5)	−0.0771 (5)	0.10664 (18)	0.0444 (11)	
C4	0.6849 (5)	0.2449 (5)	0.12284 (18)	0.0441 (11)	
C5	0.7078 (5)	0.1972 (5)	0.19220 (17)	0.0432 (11)	
C6	0.6954 (5)	0.0179 (6)	0.21040 (18)	0.0461 (11)	
C15	0.7438 (6)	0.3575 (6)	0.23320 (19)	0.0516 (14)	
C16	0.8242 (9)	0.3391 (8)	0.2982 (2)	0.080 (2)	
C41	0.8574 (5)	0.2795 (5)	0.09762 (17)	0.0444 (11)	
C42	0.9599 (6)	0.1317 (7)	0.0816 (2)	0.0585 (16)	
C43	1.1194 (6)	0.1664 (8)	0.0619 (2)	0.0673 (17)	
C44	1.1803 (6)	0.3452 (9)	0.0582 (2)	0.0661 (16)	
C45	1.0782 (6)	0.4909 (8)	0.0726 (2)	0.0617 (16)	
C46	0.9178 (6)	0.4592 (7)	0.0921 (2)	0.0548 (12)	
C61	0.7244 (7)	−0.0627 (6)	0.27457 (19)	0.0603 (14)	
H1	0.642 (7)	−0.225 (8)	0.173 (2)	0.061 (15)*	
H3	0.565 (5)	0.118 (6)	0.049 (2)	0.047 (11)*	
H4	0.61861	0.35972	0.11825	0.0528*	
H16A	0.86487	0.45818	0.31279	0.1193*	
H16B	0.91778	0.25375	0.29855	0.1193*	
H16C	0.74161	0.29367	0.32496	0.1193*	
H42	0.92086	0.01038	0.08427	0.0701*	
H43	1.18682	0.06759	0.05095	0.0807*	
H44	1.28923	0.36677	0.04607	0.0796*	
H45	1.11727	0.61201	0.06913	0.0739*	
H46	0.84985	0.55915	0.10164	0.0657*	
H61A	0.84360	−0.05933	0.28748	0.0904*	
H61B	0.68524	−0.18890	0.27422	0.0904*	
H61C	0.66278	0.00853	0.30303	0.0904*	

### Atomic displacement parameters (Å^2^)

**Table:**

	*U*^11^	*U*^22^	*U*^33^	*U*^12^	*U*^13^	*U*^23^
S2	0.0612 (7)	0.0456 (6)	0.0530 (6)	−0.0106 (5)	−0.0089 (4)	−0.0029 (4)
O15	0.128 (3)	0.0362 (17)	0.065 (2)	0.0011 (19)	−0.007 (2)	−0.0031 (14)
N1	0.070 (2)	0.0316 (17)	0.0496 (18)	−0.0030 (16)	−0.0112 (15)	0.0024 (14)
N3	0.0482 (17)	0.0417 (18)	0.0448 (17)	−0.0042 (14)	−0.0101 (13)	0.0047 (14)
C2	0.0420 (18)	0.041 (2)	0.049 (2)	−0.0012 (15)	−0.0037 (15)	−0.0004 (16)
C4	0.049 (2)	0.0365 (19)	0.0455 (19)	−0.0013 (15)	−0.0050 (15)	0.0027 (15)
C5	0.0472 (19)	0.0370 (19)	0.0447 (19)	0.0005 (15)	−0.0002 (15)	0.0012 (15)
C6	0.053 (2)	0.041 (2)	0.0433 (19)	−0.0006 (17)	−0.0027 (15)	0.0009 (16)
C15	0.069 (3)	0.036 (2)	0.050 (2)	−0.0061 (18)	0.0050 (18)	−0.0008 (16)
C16	0.123 (5)	0.055 (3)	0.058 (3)	−0.021 (3)	−0.015 (3)	−0.006 (2)
C41	0.0477 (19)	0.045 (2)	0.0390 (17)	0.0012 (16)	−0.0063 (14)	0.0027 (15)
C42	0.062 (3)	0.050 (2)	0.063 (3)	0.004 (2)	0.001 (2)	0.005 (2)
C43	0.055 (3)	0.075 (3)	0.072 (3)	0.013 (2)	0.005 (2)	−0.001 (3)
C44	0.048 (2)	0.093 (4)	0.057 (2)	−0.008 (2)	0.0027 (19)	0.005 (3)
C45	0.061 (3)	0.068 (3)	0.055 (2)	−0.014 (2)	−0.002 (2)	0.003 (2)
C46	0.060 (2)	0.052 (2)	0.052 (2)	−0.006 (2)	0.0009 (18)	0.0023 (18)
C61	0.087 (3)	0.044 (2)	0.048 (2)	−0.005 (2)	−0.007 (2)	0.0057 (18)

### Geometric parameters (Å, °)

**Table:**

S2—C2	1.689 (4)	C42—C43	1.381 (7)
O15—C15	1.214 (6)	C43—C44	1.379 (8)
N1—C2	1.368 (5)	C44—C45	1.373 (8)
N1—C6	1.394 (5)	C45—C46	1.382 (7)
N3—C2	1.318 (5)	C4—H4	0.9800
N3—C4	1.465 (5)	C16—H16A	0.9600
N1—H1	0.80 (6)	C16—H16B	0.9600
N3—H3	0.88 (4)	C16—H16C	0.9600
C4—C41	1.524 (6)	C42—H42	0.9300
C4—C5	1.531 (5)	C43—H43	0.9300
C5—C6	1.356 (6)	C44—H44	0.9300
C5—C15	1.469 (6)	C45—H45	0.9300
C6—C61	1.501 (6)	C46—H46	0.9300
C15—C16	1.499 (6)	C61—H61A	0.9600
C41—C46	1.388 (6)	C61—H61B	0.9600
C41—C42	1.396 (6)	C61—H61C	0.9600
			
S2···N3^i^	3.436 (4)	C16···H61C	2.7100
S2···H45^ii^	3.1400	C16···H61A	2.8900
S2···H16C^iii^	3.1900	C42···H16A^ix^	2.8600
S2···H3^i^	2.57 (4)	C43···H16A^ix^	3.0800
S2···H44^iv^	3.1200	C45···H61A^viii^	3.0500
O15···N1^v^	2.898 (5)	C46···H61A^viii^	3.0900
O15···C41	3.283 (5)	C61···H16B	2.7700
O15···C46	3.201 (6)	C61···H16C	2.7900
O15···C61^v^	3.333 (6)	H1···O15^vi^	2.14 (6)
O15···H1^v^	2.14 (6)	H1···H61B	2.2000
O15···H4	2.3900	H3···S2^i^	2.57 (4)
O15···H46	2.7400	H4···O15	2.3900
O15···H61B^v^	2.5400	H4···H46	2.3700
N1···O15^vi^	2.898 (5)	H16A···C42^viii^	2.8600
N3···S2^i^	3.436 (4)	H16A···C43^viii^	3.0800
N3···H42	2.7000	H16B···C6	3.0100
C2···C42	3.418 (6)	H16B···C61	2.7700
C15···C46	3.509 (6)	H16B···H61A	2.3400
C16···C61	3.033 (7)	H16C···C61	2.7900
C41···O15	3.283 (5)	H16C···H61C	2.1900
C42···C2	3.418 (6)	H16C···S2^x^	3.1900
C44···C46^vii^	3.563 (6)	H42···N3	2.7000
C44···C45^vii^	3.552 (7)	H42···C2	2.8200
C45···C46^vii^	3.571 (6)	H44···S2^iv^	3.1200
C45···C61^viii^	3.555 (6)	H45···S2^xi^	3.1400
C45···C44^vii^	3.552 (7)	H46···O15	2.7400
C45···C45^vii^	3.277 (6)	H46···H4	2.3700
C46···C15	3.509 (6)	H61A···C16	2.8900
C46···O15	3.201 (6)	H61A···H16B	2.3400
C46···C45^vii^	3.571 (6)	H61A···C45^ix^	3.0500
C46···C44^vii^	3.563 (6)	H61A···C46^ix^	3.0900
C61···C45^ix^	3.555 (6)	H61B···O15^vi^	2.5400
C61···C16	3.033 (7)	H61B···H1	2.2000
C61···O15^vi^	3.333 (6)	H61C···C15	3.0200
C2···H42	2.8200	H61C···C16	2.7100
C6···H16B	3.0100	H61C···H16C	2.1900
C15···H61C	3.0200		
			
C2—N1—C6	124.4 (3)	C44—C45—C46	120.6 (5)
C2—N3—C4	125.4 (3)	C41—C46—C45	120.6 (5)
C2—N1—H1	111 (3)	N3—C4—H4	108.00
C6—N1—H1	124 (3)	C5—C4—H4	108.00
C2—N3—H3	116 (3)	C41—C4—H4	108.00
C4—N3—H3	116 (3)	C15—C16—H16A	109.00
S2—C2—N1	119.8 (3)	C15—C16—H16B	109.00
S2—C2—N3	123.8 (3)	C15—C16—H16C	110.00
N1—C2—N3	116.4 (3)	H16A—C16—H16B	109.00
N3—C4—C5	110.0 (3)	H16A—C16—H16C	109.00
C5—C4—C41	110.1 (3)	H16B—C16—H16C	109.00
N3—C4—C41	112.4 (3)	C41—C42—H42	120.00
C4—C5—C6	119.4 (3)	C43—C42—H42	120.00
C4—C5—C15	114.5 (3)	C42—C43—H43	119.00
C6—C5—C15	126.2 (4)	C44—C43—H43	119.00
C5—C6—C61	128.7 (4)	C43—C44—H44	120.00
N1—C6—C5	118.8 (4)	C45—C44—H44	120.00
N1—C6—C61	112.4 (4)	C44—C45—H45	120.00
O15—C15—C16	118.1 (4)	C46—C45—H45	120.00
C5—C15—C16	122.8 (4)	C41—C46—H46	120.00
O15—C15—C5	119.0 (4)	C45—C46—H46	120.00
C4—C41—C42	120.9 (3)	C6—C61—H61A	110.00
C4—C41—C46	120.3 (3)	C6—C61—H61B	109.00
C42—C41—C46	118.8 (4)	C6—C61—H61C	109.00
C41—C42—C43	119.7 (5)	H61A—C61—H61B	109.00
C42—C43—C44	121.1 (5)	H61A—C61—H61C	109.00
C43—C44—C45	119.2 (5)	H61B—C61—H61C	109.00
			
C6—N1—C2—S2	−167.7 (3)	C4—C5—C6—N1	−5.6 (6)
C6—N1—C2—N3	10.2 (6)	C4—C5—C6—C61	175.5 (4)
C2—N1—C6—C5	−12.3 (6)	C15—C5—C6—N1	175.0 (4)
C2—N1—C6—C61	166.8 (4)	C15—C5—C6—C61	−3.9 (7)
C4—N3—C2—S2	−171.4 (3)	C4—C5—C15—O15	17.8 (6)
C4—N3—C2—N1	10.9 (6)	C4—C5—C15—C16	−159.8 (5)
C2—N3—C4—C5	−25.7 (5)	C6—C5—C15—O15	−162.8 (5)
C2—N3—C4—C41	97.4 (4)	C6—C5—C15—C16	19.6 (7)
N3—C4—C5—C6	22.0 (5)	C4—C41—C42—C43	−176.7 (4)
N3—C4—C5—C15	−158.6 (3)	C46—C41—C42—C43	1.3 (6)
C41—C4—C5—C6	−102.5 (4)	C4—C41—C46—C45	176.3 (4)
C41—C4—C5—C15	77.0 (4)	C42—C41—C46—C45	−1.6 (6)
N3—C4—C41—C42	−42.5 (5)	C41—C42—C43—C44	0.5 (7)
N3—C4—C41—C46	139.6 (4)	C42—C43—C44—C45	−2.0 (7)
C5—C4—C41—C42	80.5 (4)	C43—C44—C45—C46	1.6 (7)
C5—C4—C41—C46	−97.4 (4)	C44—C45—C46—C41	0.2 (7)

Symmetry codes: (i) −*x*+1, −*y*, −*z*; (ii) *x*−1, *y*−1, *z*; (iii) −*x*+1, *y*−1/2, −*z*+1/2; (iv) −*x*+2, −*y*, −*z*; (v) *x*, *y*+1, *z*; (vi) *x*, *y*−1, *z*; (vii) −*x*+2, −*y*+1, −*z*; (viii) −*x*+2, *y*+1/2, −*z*+1/2; (ix) −*x*+2, *y*−1/2, −*z*+1/2; (x) −*x*+1, *y*+1/2, −*z*+1/2; (xi) *x*+1, *y*+1, *z*.

### Hydrogen-bond geometry (Å, °)

**Table:**

*D*—H···*A*	*D*—H	H···*A*	*D*···*A*	*D*—H···*A*
N1—H1···O15^vi^	0.80 (6)	2.14 (6)	2.898 (5)	158 (5)
N3—H3···S2^i^	0.88 (4)	2.57 (4)	3.436 (4)	168 (4)
C61—H61B···O15^vi^	0.96	2.54	3.333 (6)	140

Symmetry codes: (vi) *x*, *y*−1, *z*; (i) −*x*+1, −*y*, −*z*.
